# Three new species of the millipede genus *Hyleoglomeris* Verhoeff, 1910 from the Aegean region of Greece (Diplopoda, Glomerida, Glomeridae)

**DOI:** 10.3897/BDJ.1.e1000

**Published:** 2013-11-06

**Authors:** Sergei Golovatch

**Affiliations:** † Russian Academy of Sciences, Moscow, Russia

**Keywords:** Millipede, *Hyleoglomeris*, new species, cave, Chios, Rhodes, Kalymnos, Greece

## Abstract

Three new cavernicolous species of *Hyleoglomeris* are described from Greece: *Hyleoglomeris subreducta* sp.n., from Chios Island, *Hyleoglomeris translucida* sp.n., from Rhodes Island, and *Hyleoglomeris insularis* sp.n., from Kalymnos Island, all in the Aegean Sea.

## Introduction

In Eurasia, the largely Holarctic, warm temperate to tropical millipede order Glomerida is long known to show two main centres of generic and species diversification, one in the Mediterranean, the other in the Oriental realm (Golovatch et al. 2010). *Hyleoglomeris* Verhoeff, 1910 is the only genus shared by the two, being the largest and certainly the most widespread in the entire order. It dominates the Oriental fauna of Glomerida, containing nearly 100 nominate species ranging from Serbia, Balkans in the West to Japan in the East, and the Sunda Archipelago (Sulawesi) in the Southeast (Golovatch et al. 2006, Golovatch et al. 2012, Golovatch et al. 2013, Golovatch and Geoffroy 2012, Makarov et al. 2013). In the Balkans and northwestern Anatolia, the range slightly overlaps with that of the Euro-Mediterranean and also speciose genus *Glomeris* Latreille, 1803. In this overlap area, all known *Glomeris* species seem to be epigean, whereas the *Hyleoglomeris* spp. appear to be subterranean. Both these genera are deemed quite closely related, at least belonging to the same subfamily Glomerinae Leach, 1815 (see Hoffman 1980), even though Mauriès (2006) places them in different tribes. The main distinction between *Glomeris* and *Hyleoglomeris* lies in the considerably less strongly differentiated caudofemoral process on male leg-pair 19 (= telopods) in the former genus (Mauriès 1971). In *Hyleoglomeris*, this outgrowth has become enlarged, set at nearly a right angle to the femur and directed more ventrally than mesally, with the tip supporting a membranous sac. *Glomeris* appears to be simpler, likely more basal, in that its species are normally larger in size, with less numerous striae on the second tergite (often referred to as a thoracic shield, in any event to be treated only as a highly conventional term), in possessing a less strongly reduced male leg-pair 17, and usually in having no caudal tubercle at the base of the tibial outgrowth of the telopod (Golovatch et al. 2012).

Greece, both mainland and islands, currently supports seven genera and 11 species of Glomerida (Thaler 2000, Thaler 2003), of which two species belong to *Hyleoglomeris*: *Hyleoglomeris beroni* Mauriès, 1984, from a cave on Naxos Island, and *Hyleoglomeris epirotica* (Mauriès, 1966), from a cave near Ioannina, Epirus (Mauriès 1966, Mauriès 1984). The present note puts on record another three new species of this very large genus, each found in a cave on three other Greek islands in the Aegean Sea. A rich fauna of *Hyleoglomeris*, especially of cavernicoles, is evidently present at or near the western periphery of its distribution area.

## Materials and methods

The material underlying this contribution was received for study through the courtesy of Pavel Stoev, of the National Museum of Natural History, Sofia, Bulgaria (NMNHS). All samples had been taken by Petar Beron (NMNHS), an outstanding collector and researcher. Most of the types have been returned to the NMNHS collection, with only a few paratypes retained for the collection of the Zoological Museum, State University of Moscow, Russia (ZMUM), as indicated below.

## Taxon treatments

### Hyleoglomeris subreducta

Golovatch, 2013 sp. n.

urn:lsid:zoobank.org:act:66006F7F-BDDD-417E-92DF-34B896F75A5B

#### Materials

- **Type status:** Holotype. **Occurrence:** recordedBy: P. Beron; sex: 1 male; **Location:** island: Chios; country: Greece; verbatimLocality: village Haghios Galos (Agiongalas, Haghia Gala), 65 km from town of Chios, Cave Hagiogalousaina; **Event:** eventDate: 1987-05-12; **Record Level:** institutionCode: NMNHS
- **Type status:** Paratype. **Occurrence:** recordedBy: P. Beron; sex: 1 male, 3 female, 3 juveniles; **Location:** island: Chios; country: Greece; verbatimLocality: village Haghios Galos (Agiongalas, Haghia Gala), 65 km from town of Chios, Cave Hagiogalousaina; **Event:** eventDate: 1987-05-12; **Record Level:** institutionCode: NMNHS
- **Type status:** Paratype. **Occurrence:** recordedBy: P. Beron; sex: 1 male, 1 female; **Location:** island: Chios; country: Greece; verbatimLocality: village Haghios Galos (Agiongalas, Haghia Gala), 65 km from town of Chios, Cave Hagiogalousaina; **Event:** eventDate: 1987-05-12; **Record Level:** institutionCode: ZMUM

#### Description

Length of holotype ca 5.5 mm, width (maximum on tergum 2) ca 2.5 mm; length of paratypes ca 5.0-6.0 mm, width on tergum 2 ca 2.1-3.0 mm, or males and females, respectively. Body from nearly entirely pallid (especially so in smaller specimens) to coloration remnants persisting on head and terga (Fig. 1a, b, c). Head usually with a considerably infuscate, large, brownish to blackish patch around Tömösváry’s organ and ocelli each side (Fig. 1b, c), antennae pallid to very faintly brownish yellow. Trunk from entirely pallid to very faintly brown or grey-brown, in the latter case with a peculiar pattern (Fig. 1a, b, c). Collum very slightly marbled brownish on sides and with a rather wide, light brownish, subcaudal band in front of a narrowly flavous caudal margin (Fig. 1a, b). Tergum 2 (= thoracic shield) with a similar pattern, but subcaudal band wider, slightly broadened and remaining only above schism laterally while central part subtriangular and extending nearly up to front margin (Fig. 1a, b, c). Following terga with a similar pattern as well, each with a paramedian pair of large, transversely oval, flavous, sublateral, anterior spots above broadly flavous lateral margin. Last tergum (= pygidium) nearly entirely and uniformly light brown or grey-brown, with a broad flavous band at caudal margin (Fig. 1a, b, c). Ocelli 5+1 or 6+1, convex, completely translucid, but mostly clearly discernible due to an infuscated nearby background (Fig. 1b, c). Tömösváry’s organ pallid, transverse-oval, ca 1.4 times wider than long. Antennomere 6 rather long, ca 2.0–2.1 times as long as high. Collum with two transverse striae. Tergum 2 with a narrow hyposchism extending behind to reach the caudal tergal margin; 7-8 superficial transverse striae, only one starting below schism, one level with, all others above schism while three (never last one from below) crossing the dorsum. Male anal shield regularly rounded at caudal margin. Male leg 17 (Fig. 2a) with a rather high, regularly rounded, outer coxal lobe; telopodite 4-segmented, tarsus with three strong apical spines. Male leg 18 (Fig. 2b) with a regularly rounded syncoxital notch; telopodite 4-segmented, tarsus with one apical spine. Male legs 19, or telopods (Fig. 2c), with a high, regularly rounded, central syncoxital lobe flanked by two setose horns, each latter only slightly higher than central lobe and crowned by a minute, elongate, acuminate, membranous lobule devoid of adjacent structures. Prefemur and, to a lesser extent and only parabasally, femur micropapillate laterally. Caudomedial femoral process prominent, directed distomedially at ca 100º to femur, mostly strongly chitinized, only apically with a small membranous sac, but devoid of any chitinized lobe. Caudomedial process of tibia evident, sac-shaped, membranous, with an evident, rounded tubercle on caudal face at base. Tarsus rather strongly curved, subacuminate apically.

#### Diagnosis

Differs from congeners in a partly to completely unpigmented body, coupled with sometimes still persisting remnants of a peculiar colour pattern, a rather long antennomere 6 which is ca 2.0–2.1 times as long as high, as well as by a narrow hyposchism which only reaches the caudal margin of tergum 2, and 7-8 transverse striae of which three cross the dorsum on tergum 2. Differs clearly from all known Greek congeners, including two new ones described below, also by a 4-segmented male telopodite 17 (Fig. 2a).

#### Etymology

To emphasize the nearly fully to fully reduced body coloration similar to the condition observed in the southern Chinese cavernicole, *Hyleoglomeris reducta* Golovatch, Geoffroy & Mauriès, 2006. An adjective.

#### Taxon discussion

Due to such a troglomorphic feature as the completely or nearly completely unpigmented body, this species may well prove to be a troglobite. This cave on Chios is known to harbour at least one more endemic troglobite, the false-scorpion *Chthonius chius* Schawaller, 1990 (Pseudoscorpiones, Chthoniidae) (Harvey 2008).

### Hyleoglomeris translucida

Golovatch, 2013 sp. n.

urn:lsid:zoobank.org:act:85DE9877-212A-4767-87B8-90D145E27B25

#### Materials

- **Type status:** Holotype. **Occurrence:** recordedBy: P. Beron; sex: 1 male; **Location:** island: Rhodes; country: Greece; verbatimLocality: village Archangelos, Cave Coumellos; **Event:** eventDate: 1987-05-02; **Record Level:** institutionCode: NMNHS
- **Type status:** Paratype. **Occurrence:** recordedBy: P. Beron; sex: 2 males, 6 females, 2 juveniles; **Location:** island: Rhodes; country: Greece; verbatimLocality: village Archangelos, Cave Coumellos; **Event:** eventDate: 1987-05-02; **Record Level:** institutionCode: NMNHS
- **Type status:** Paratype. **Occurrence:** recordedBy: P. Beron; sex: 1 male, 1 female; **Location:** island: Rhodes; country: Greece; verbatimLocality: village Archangelos, Cave Coumellos; **Event:** eventDate: 1987-05-02; **Record Level:** institutionCode: ZMUM

#### Description

**Figure 3.**
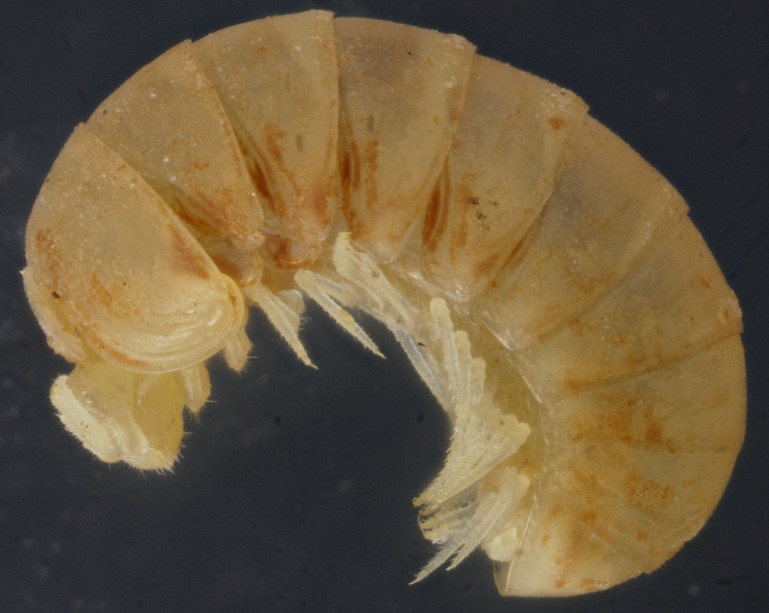
Habitus of *Hyleoglomeris translucida* sp. n., female paratype, lateral view. Photo by K. Makarov, not taken to scale.

Length of holotype ca 4.1 mm, width (maximum on tergum 2) ca 1.8 mm; length of paratypes ca 4.0-4.3 mm, width on tergum 2 ca 1.8-2.0 mm, or 4.3-5.0 and 2.0-2.6 mm in males and females, respectively. Body entirely pallid (Fig. 3).

Ocelli ca 6+1, convex, completely translucid, poorly discernible (Fig. 3). Tömösváry’s organ pallid, transverse-oval, ca 1.4-1.5 times wider than long. Antennomere 6 rather long, ca 2.1–2.2 times as long as high.

Collum with two transverse striae. Tergum 2 with a narrow hyposchism extending behind to reach the caudal tergal margin; 6-7 superficial transverse striae, two starting below schism, one level with, all others above schism, with only one (never last one from below) crossing the dorsum. Male anal shield regularly rounded at caudal margin.

Male leg 17 (Fig. 4a) with a rather low, regularly rounded, outer coxal lobe; telopodite 3-segmented, tarsus with two strong apical spines.

Male leg 18 (Fig. 4b) with a narrow syncoxital notch; telopodite 4-segmented, tarsus with one apical spine.

Telopods (Fig. 4c, d) with a medium-sized, regularly rounded, central syncoxital lobe flanked by two setose horns, each latter clearly higher than central lobe and crowned by a small, elongate membranous lobule devoid of adjacent structures. Prefemur and, to a lesser extent and only parabasally, femur micropapillate laterally. Caudomedial femoral process prominent, directed distomedially at ca 100º to femur, mostly strongly chitinized, only apically with a small membranous sac, on caudal face slightly setose. Caudomedial process of tibia evident, sac-shaped, membranous, at base with an evident, parabasally poorly setose tubercle on caudal face. Tarsus rather strongly curved, subacuminate apically.

#### Diagnosis

Differs from congeners in a completely unpigmented body, coupled with a rather long antennomere 6 which is ca 2.1–2.2 times as long as high, as well as by a narrow hyposchism which only reaches the caudal margin of tergum 2, 6-7 transverse striae of which only 1-2 cross the dorsum on tergum 2, a 3-segmented male telopodite 17, and a caudally slightly setose distomesal process of the telopod femur.

#### Etymology

To emphasize the fully unpigmented, translucid body. An adjective.

#### Taxon discussion

Due to such a clearly troglomorphic feature as the completely unpigmented body, this species may prove to be a troglobite.

### Hyleoglomeris insularis

Golovatch, 2013 sp. n.

urn:lsid:zoobank.org:act:87F46896-93D4-49A2-A857-C06F44A8910A

#### Materials

- **Type status:** Holotype. **Occurrence:** recordedBy: P. Beron; sex: 1 male; **Location:** island: Kálimnos; country: Greece; verbatimLocality: village Scalia, Cave Scalia; **Event:** eventDate: 1987-05-04; **Record Level:** institutionCode: NMNHS
- **Type status:** Paratype. **Occurrence:** recordedBy: P. Beron; sex: 2 males, 2 females; **Location:** island: Kálimnos; country: Greece; verbatimLocality: village Scalia, Cave Scalia; **Event:** eventDate: 1987-05-04; **Record Level:** institutionCode: NMNHS
- **Type status:** Paratype. **Occurrence:** recordedBy: P. Beron; sex: 1 male, 1 female; **Location:** island: Kálimnos; country: Greece; verbatimLocality: village Scalia, Cave Scalia; **Event:** eventDate: 1987-05-04; **Record Level:** institutionCode: ZMUM

#### Description

Length of holotype ca 6.0 mm, width (maximum on tergum 2) ca 3.0 mm; length of paratypes ca 6.0-6.2 mm, width on tergum 2 ca 3.0-3.1 mm, or 6.2-7.5 and 3.2-3.4 mm in males and females, respectively. Body nearly entirely pallid (Fig. 5), only dorsal side of head retaining a faint to mediocre, rather uniform brownish coloration often growing a little darker on antennae and pale grey to nearly blackish around ocelli (Fig. 5b).

Ocelli 6+1 or perhaps 7+1, convex, completely translucid, but mostly clearly discernible due to an infuscated nearby background (Fig. 5b). Tömösváry’s organ pallid, transverse-oval, ca 1.4-1.5 times wider than long. Antennomere 6 long, ca 2.3–2.4 times as long as high.

Collum with two transverse striae. Tergum 2 with a rather broad hyposchism extending considerably behind caudal tergal margin (Fig. 5a); 4-5 superficial transverse striae, 1-2 starting below schism, remaining 2-3 above it, with three (never last one from below) crossing the dorsum. Male anal shield regularly rounded at caudal margin.

Male leg 17 (Fig. 6a) with a rather low, regularly rounded, outer coxal lobe; telopodite 3-segmented, tarsus with two strong apical spines.

Male leg 18 (Fig. 6b) with a narrow syncoxital notch; telopodite 4-segmented, tarsus with one apical spine.

Telopods (Fig. 6c) with a high, rounded, clearly emarginate, central syncoxital lobe flanked by two setose horns, each latter only slightly higher than central lobe and crowned by a minute, elongate, acute, membranous lobule devoid of adjacent structures. Only prefemur micropapillate laterally. Caudomedial femoral process prominent, directed distomedially at ca 100º to femur, mostly strongly chitinized, only apically with a small membranous sac, but devoid of any chitinized lobe. Caudomedial process of tibia evident, sac-shaped, membranous, with an evident, rounded tubercle on caudal face at base. Tarsus rather modestly curved, subacuminate apically.

#### Diagnosis

Differs from congeners in a partly unpigmented body with only the head retaining some pigment, coupled with a long antennomere 6 which is ca 2.3–2.4 times as long as high, as well as by a rather broad hyposchism produced considerably behind the caudal margin of tergum 2, and only 4-5 transverse striae, of which three cross the dorsum on tergum 2.

#### Etymology

To emphasize the provenance from an island. An adjective.

#### Taxon discussion

Due to such a troglomorphic feature as a nearly completely unpigmented body, this species may well prove to be a troglobite. This cave on Kalimnos is known to support at least one more endemic troglobite, the woodlouse *Cordioniscus kalimnosi* Andreev, 1997 (Isopoda, Oniscidea, Styloniscidae) (Schmalfuss 2003).

## Supplementary Material

XML Treatment for Hyleoglomeris subreducta

XML Treatment for Hyleoglomeris translucida

XML Treatment for Hyleoglomeris insularis

## Figures and Tables

**Figure 1a.**
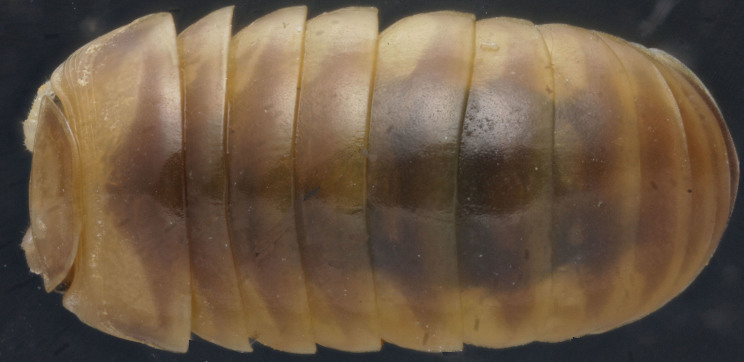
Dorsal view.

**Figure 1b.**
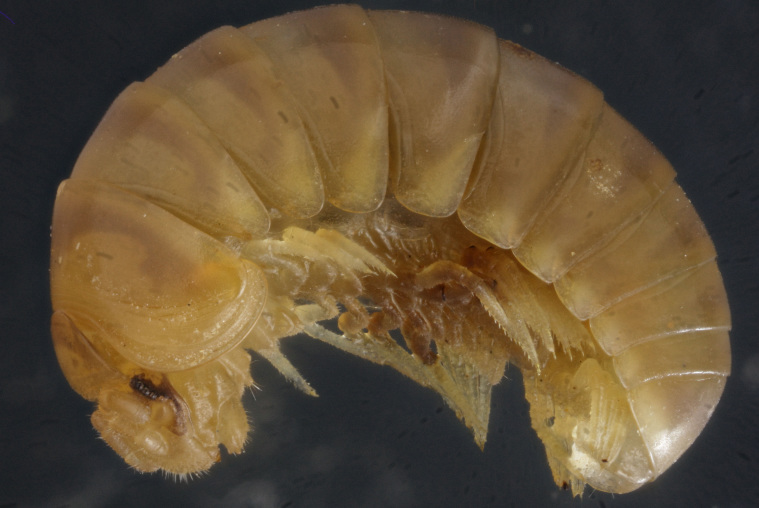
Lateral view.

**Figure 1c.**
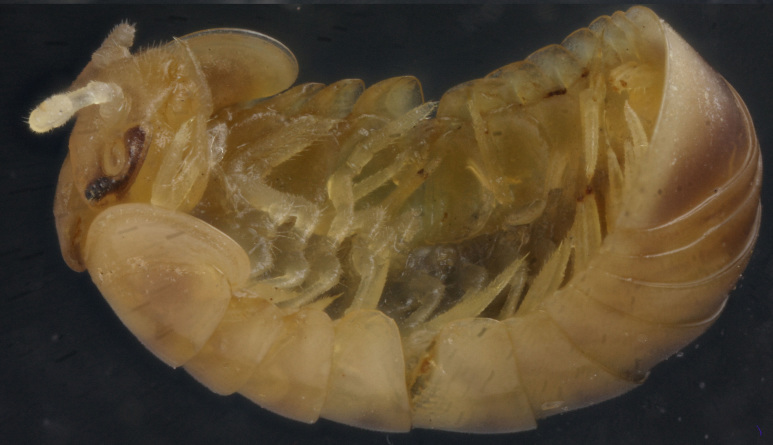
Ventrolateral view.

**Figure 2a.**
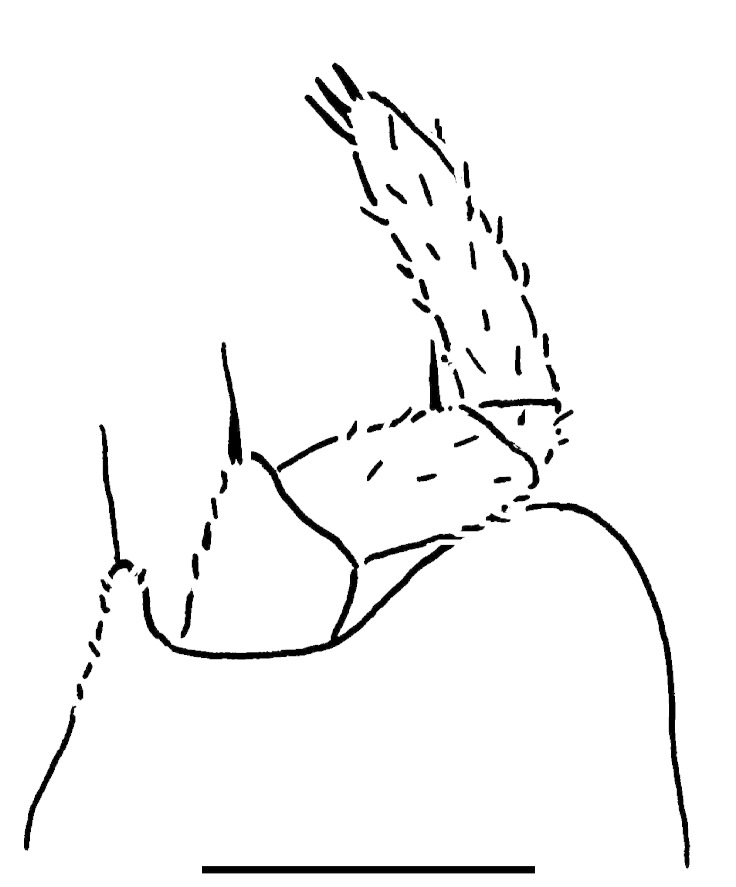
Leg 17, front view. Scale bar: 0.2 mm.

**Figure 2b.**
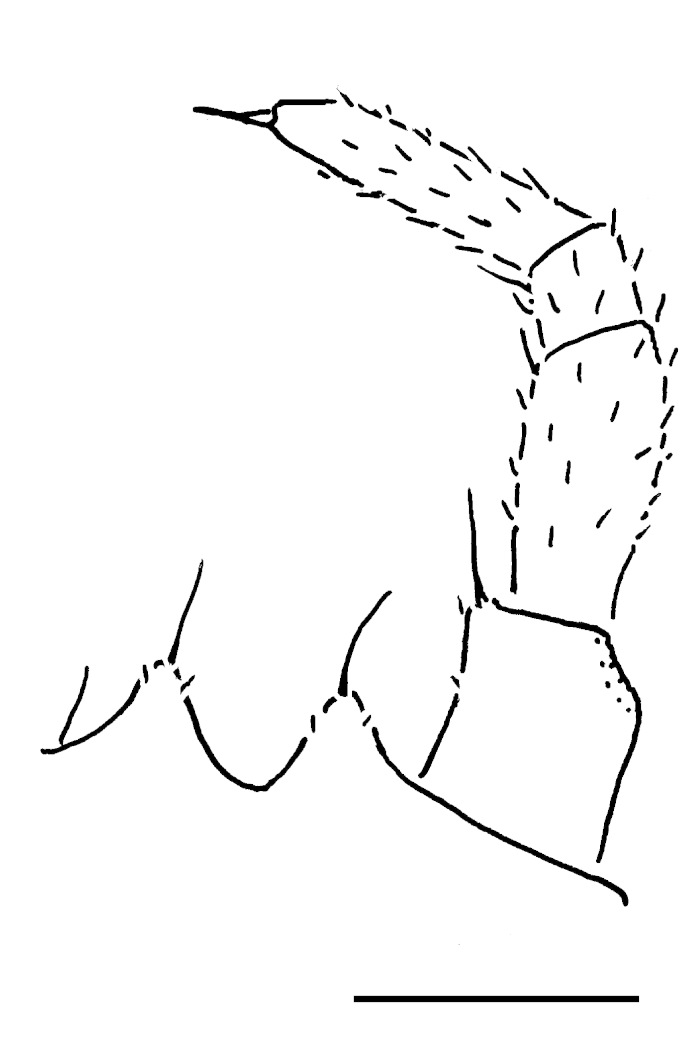
Leg 18, front view. Scale bar: 0.2 mm.

**Figure 2c.**
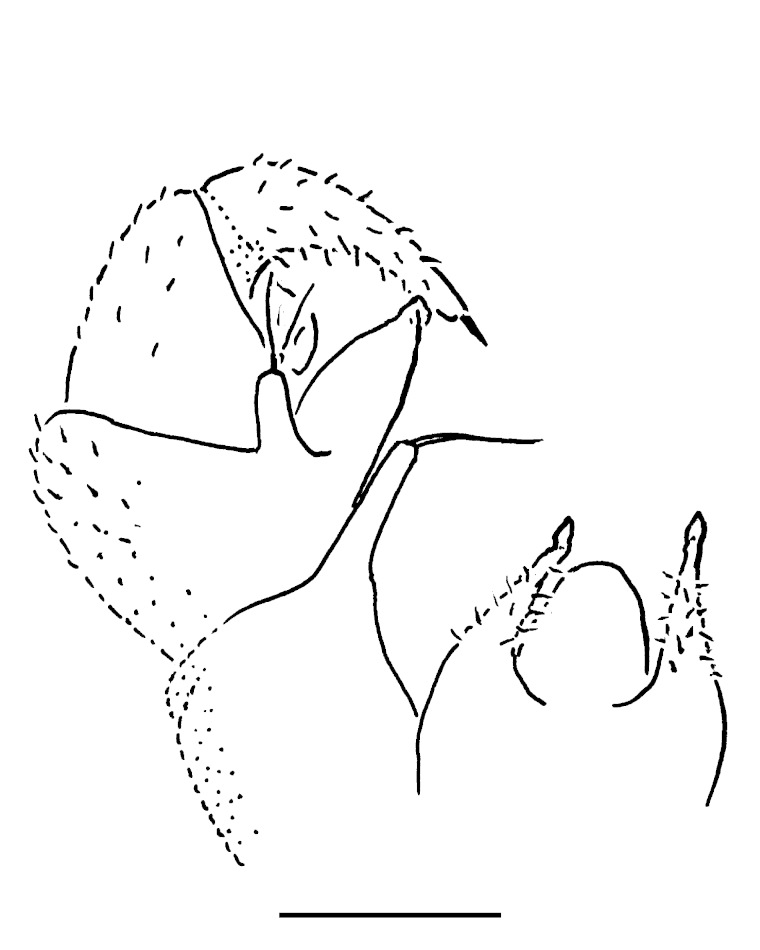
Leg 19, front view. Scale bar: 0.2 mm.

**Figure 4a.**
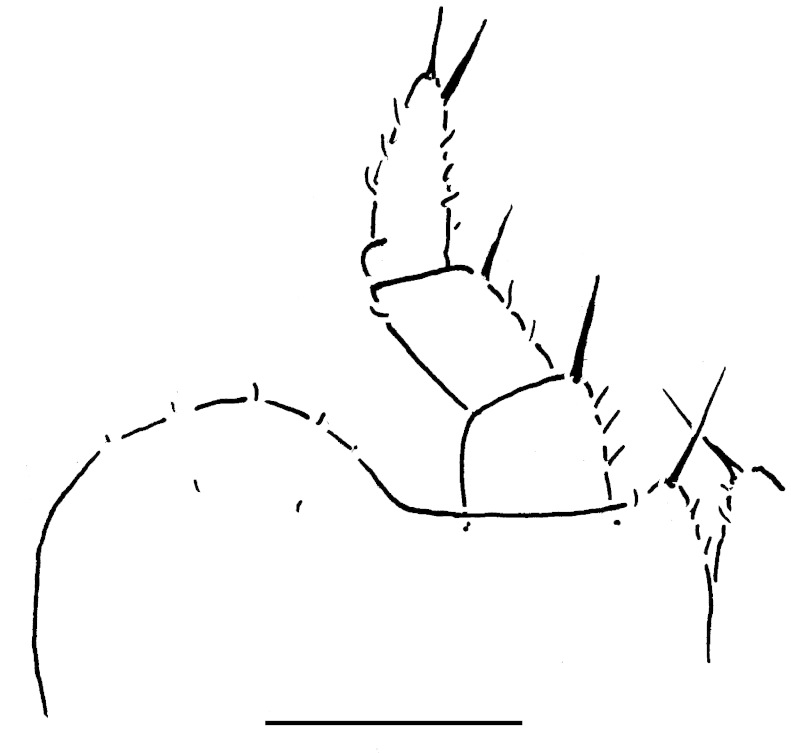
Leg 17, front view. Scale bar: 0.1 mm.

**Figure 4b.**
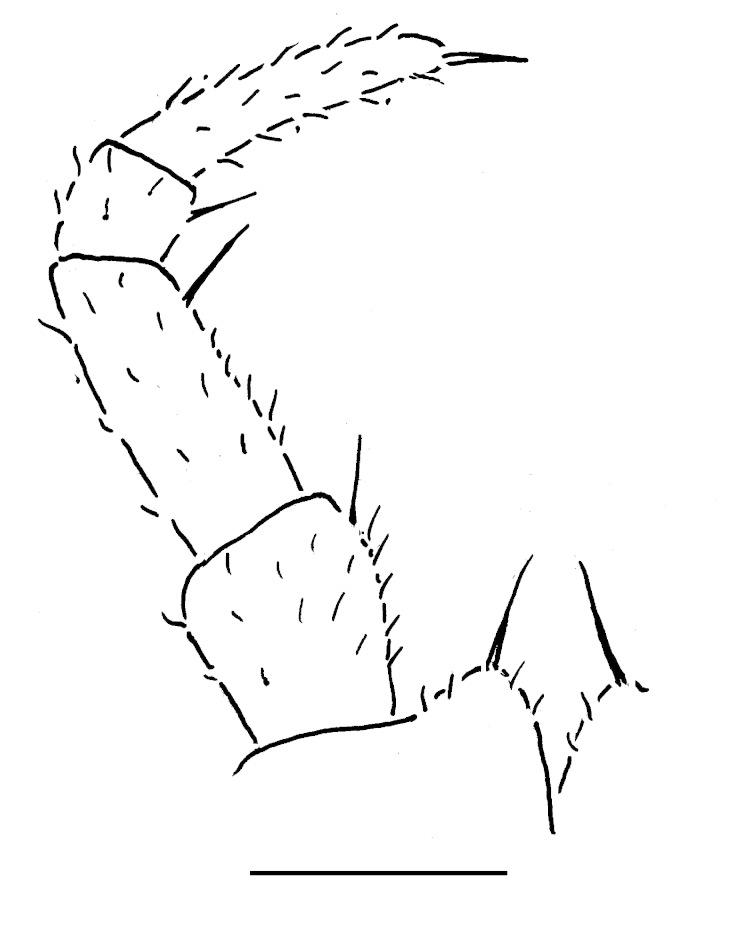
Leg 18, front view. Scale bar: 0.1 mm.

**Figure 4c.**
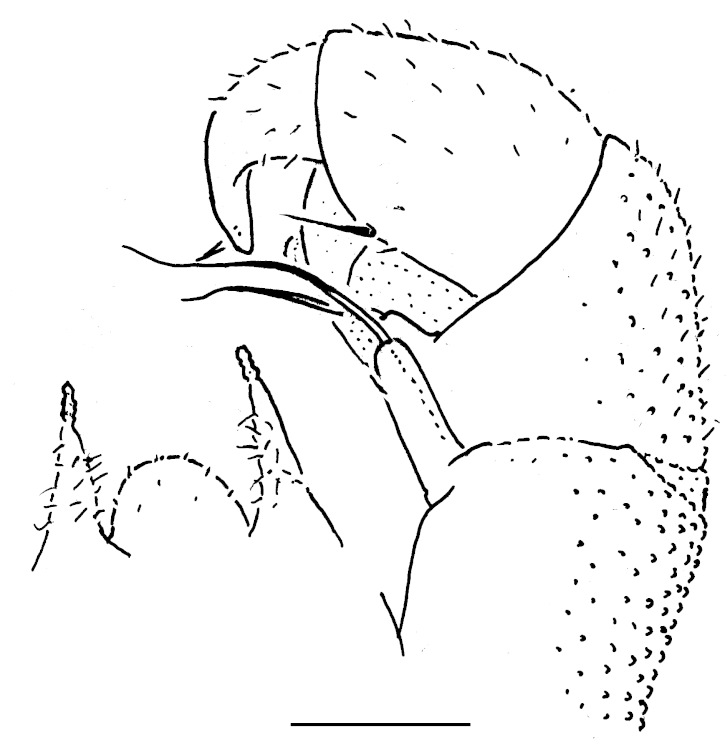
Leg 19, front view. Scale bar: 0.1 mm.

**Figure 4d.**
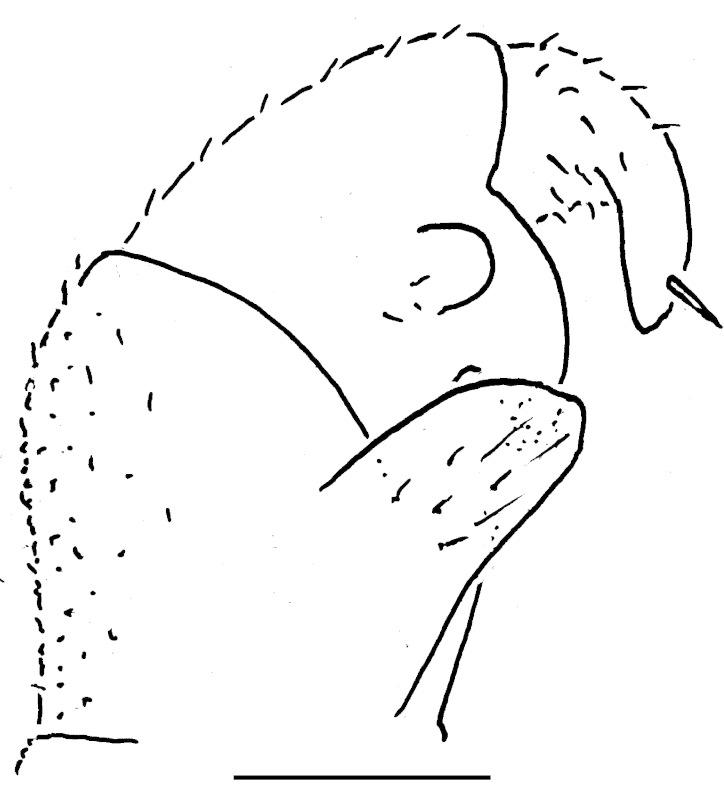
Leg 19, caudal view. Scale bar: 0.1 mm.

**Figure 5a.**
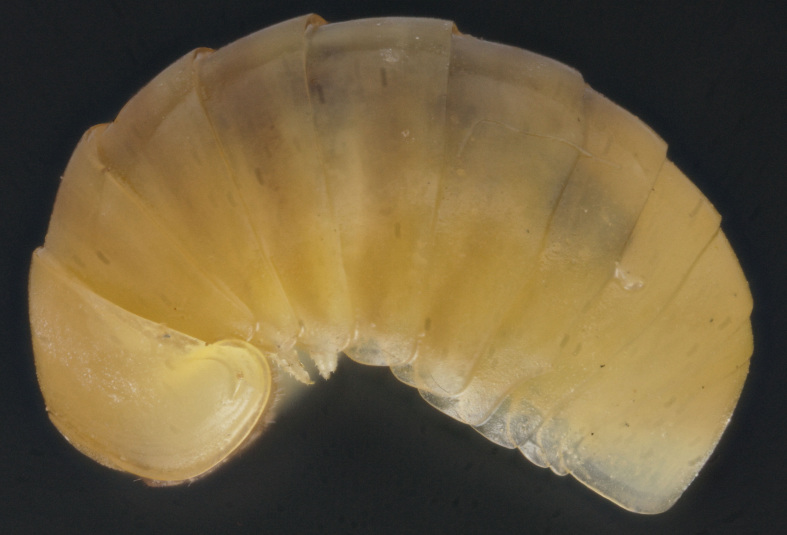
Lateral view.

**Figure 5b.**
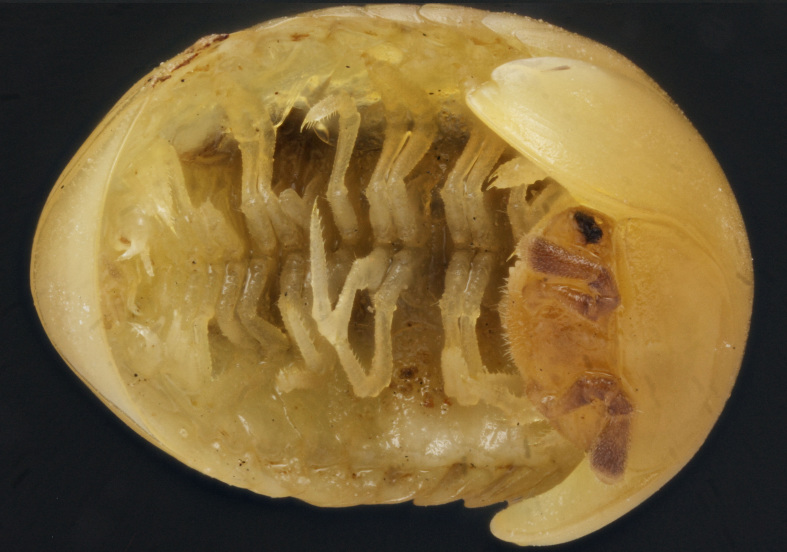
Ventral view.

**Figure 6a.**
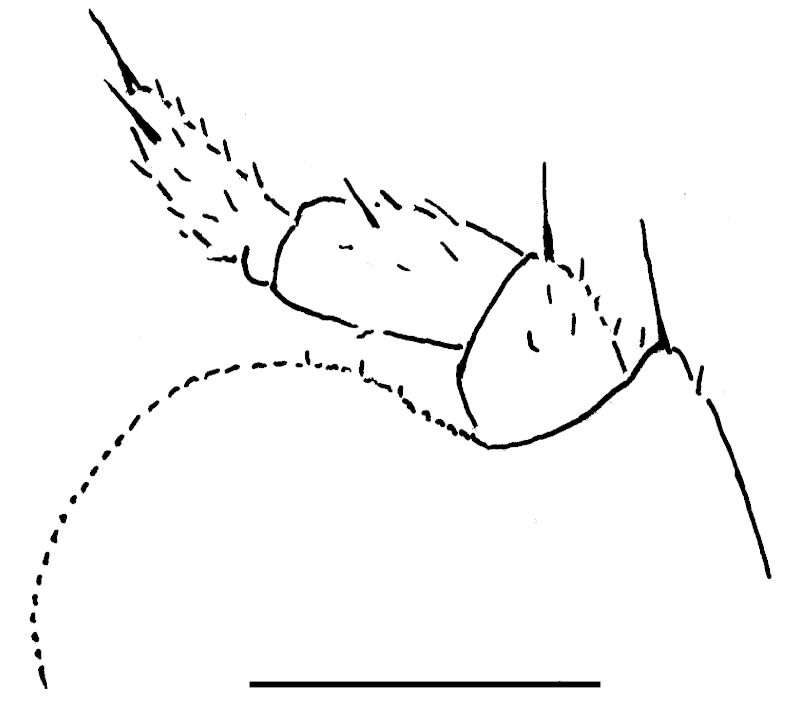
Leg 17. Scale bar: 0.2 mm.

**Figure 6b.**
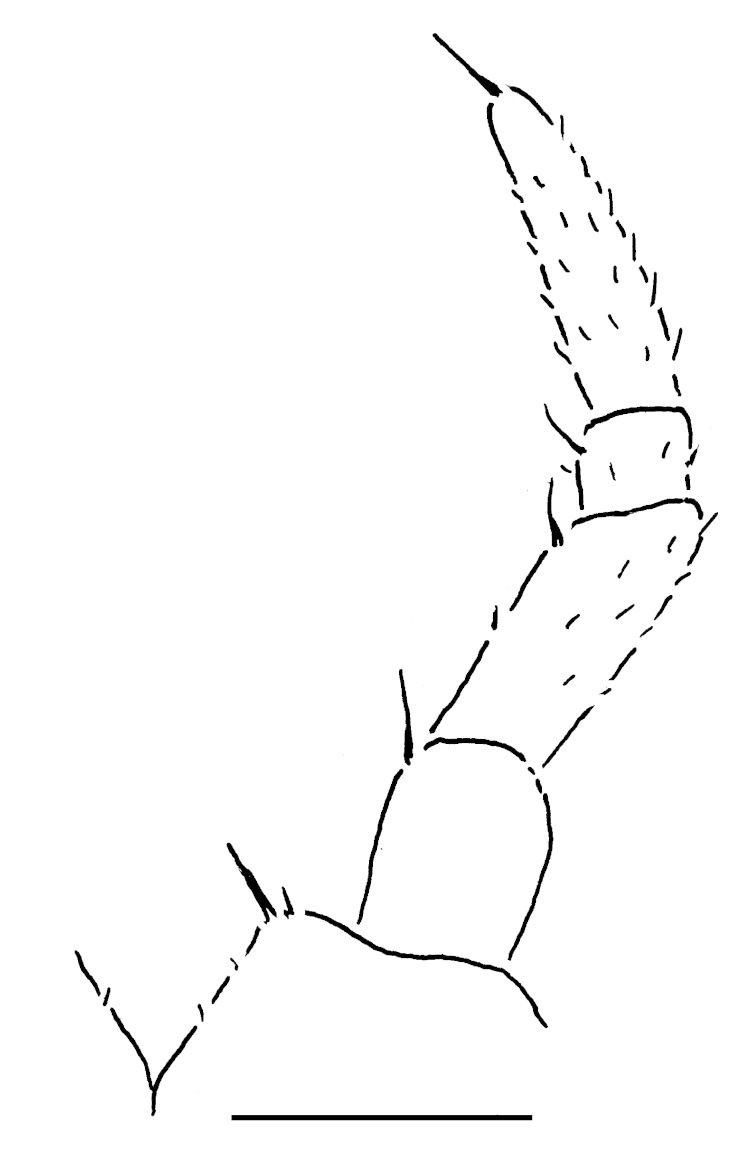
Leg 18. Scale bar: 0.2 mm.

**Figure 6c.**
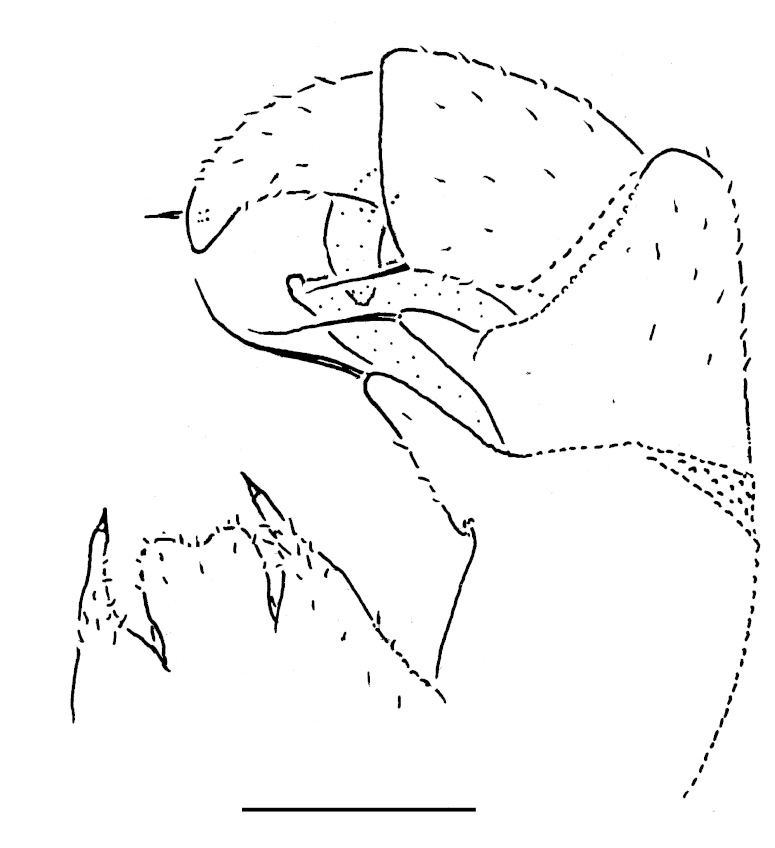
Leg 19. Scale bar: 0.2 mm.
